# Public variant databases: liability?

**DOI:** 10.1038/gim.2016.189

**Published:** 2016-12-15

**Authors:** Adrian Thorogood, Robert Cook-Deegan, Bartha Maria Knoppers

**Affiliations:** 1 Centre of Genomics and Policy, McGill University, Montreal, Quebec, Canada; 2 School for the Future of Innovation in Society, Arizona State University, and Consortium for Science, Policy & Outcomes, Tempe, Arizona, USA; 3 FasterCures, a Center of the Milken Institute, Washington, DC, USA; 4 Centre of Genomics and Policy, McGill University, Montreal, Quebec, Canada

**Keywords:** data sharing, genomics, legal, liability, variant databases

## Abstract

Public variant databases support the curation, clinical interpretation, and sharing of genomic data, thus reducing harmful errors or delays in diagnosis. As variant databases are increasingly relied on in the clinical context, there is concern that negligent variant interpretation will harm patients and attract liability. This article explores the evolving legal duties of laboratories, public variant databases, and physicians in clinical genomics and recommends a governance framework for databases to promote responsible data sharing.

*Genet Med* advance online publication 15 December 2016

Human variant databases support the aggregation, curation, and sharing of data on disease-associated variants.^1,2,3,4^ Variant databases not only curate the literature but also facilitate access to unpublished variant classifications generated in diagnostic laboratories. Making this information available to laboratories, clinicians, and patients supports accurate and timely diagnosis, which in turn improves clinical outcomes. Indeed, public variant databases are increasingly relied on during genomic testing to clarify the clinical significance of variants in support of diagnosis or targeted treatment.^5^ For clinically oriented databases, harm to patients resulting from misinterpretation is a central ethical and legal concern. Previous ethical/legal discussion concerning variant databases has focused on other concerns such as privacy, commercialization, and database sustainability.^6,7,8^ Building on existing guidance, we outline the ethical—and potentially legal—duties of databases to ensure the quality, accuracy, and currency of variant data and recommend best practices to manage legal risk through transparency and contractual frameworks.

The BRCA Exchange Web Portal (http://brcaexchange.org/) is used as a case study. Certain variants in the *BRCA1/BRCA2* genes are “pathogenic”—meaning they disrupt protein function and increase the risk of developing cancer—whereas others are benign or of unknown significance. The BRCA Exchange is a publicly available assembly of these *BRCA1/BRCA2* variant classifications. It has two publicly accessible tiers:

1. Consensus Space: publicly shares consensus variant classifications curated by an international expert panel
2. Public Research Space: publicly shares a wide range of *BRCA* variants and classifications available from multiple databases not yet curated by the expert panel

A third tier, “Curation Space,” is undergoing development and will facilitate expert curation of individual (case)-level data by credentialed users.

The BRCA Exchange Web Portal is the first product of the BRCA Challenge, an international collaboration to improve our understanding of the genetic causes of breast and ovarian cancer and to make this information publicly available and easily accessible. The BRCA Exchange is supported by the Global Alliance for Genomics and Health (GA4GH), an international coalition of more than 400 research institutions, health centers, patient groups, and life science and information technology companies. The goals of GA4GH are to enable effective and responsible sharing of genomic and clinical data and to support projects that demonstrate the value of data sharing.^9^

We begin by reviewing a 2016 lawsuit (*Williams v. Athena*) in the United States involving allegations of negligent variant interpretation by a genetic testing laboratory. Because the outcome of this case is not yet known, our discussion is based largely on allegations made by the plaintiff. These claims have not yet been proven in court. Nonetheless, this case illustrates the kinds of patient harms and liability concerns that may arise in the context of genomic testing and variant interpretation. Variant databases now complement the efforts of laboratories to curate and interpret variants. Curation is the collection (from various sources), annotation, and maintenance of variant data.^10^ Interpretation is the evaluation of the evidence regarding a linkage between a genetic variant and a disease or condition and making an assertion about that linkage (or lack thereof).^10^ In light of *Williams v. Athena*, might databases also have legal duties toward patients to ensure the quality and currency of variant data? What ethical, and potentially legal, standards apply to their activities? Finally, what steps can variant databases take to ensure that laboratories submit high-quality data and that physicians use databases responsibly?

## *Williams V. Athena*: Negligent Variant Interpretation?

The facts alleged in *Williams v. Athena*—a lawsuit pending in a South Carolina federal court—illustrate the serious harm that could potentially arise from variant misinterpretation. Because the outcome of this case is not yet known, our discussion is largely based on claims made by the plaintiff.^11^ The case concerns the death of Christian Jacob Millare, born in 2005. The plaintiff alleges the following events occurred: Christian started to have seizures after vaccination at 4 months and was prescribed a standard treatment of anticonvulsants. In 2007, Christian’s DNA was submitted to the Athena laboratory to test him for Dravet syndrome, a severe form of epilepsy associated with mutations in the *SCN1A* gene. A positive result would have led to a change in treatment because conventional anticonvulsants exacerbate seizures in patients with Dravet syndrome. The 2007 test report from Athena concluded that Christian had a “variant of unknown significance.” Christian continued to receive the standard treatment until experiencing a fatal seizure in January 2008. It was only in 2015 that the laboratory company issued a revised report listing Christian’s variant as pathogenic (without citing new evidence). In the resulting wrongful death lawsuit, the plaintiff alleges that Athena negligently interpreted the clinical validity of Christian’s *SCNA1* mutation.

A central issue in this case is who is legally responsible for variant interpretation: the laboratory or the physician?^12^ A laboratory’s traditional responsibility is for the accuracy of the test, including the handling of samples and reporting of results. Physicians are responsible for interpreting the clinical significance of a test result in the context of a particular patient. To do so, a physician needs to remain current regarding the meaning and limitations of test results. However, this division of responsibility between physicians and laboratories has increasingly blurred in the genomics age.^13^ Laboratories are now expected to aid in variant interpretation and to update patients or their physicians when interpretations change. Current regulations and guidelines require laboratories and their directors to provide physicians with adequate documentation to support interpretation, such as the level of existing knowledge of the variants and a description of the supporting evidence^14,15^ (42 CFR 493.1445(e)(8)). Some suggest that the duties of physicians should be limited to reasonable interpretations based on the laboratory report.^16^ In fact, it is difficult to conceive how any laboratory or physician alone can stay abreast of the current genomic state of knowledge. Variant databases support variant interpretation but further blur respective responsibilities between submitting laboratories, variant databases, and physicians.

An additional challenge raised by this case is how to determine the legal standard that applies to variant interpretation. Note that the legal standard of care is judged at the time when an alleged fault is committed. The Clinical Laboratory Improvement Amendments regulations that govern clinical laboratories in the United States address analytic validity but do not impose mandatory standards for determining the clinical validity of a particular genetic test.^17^ The American College of Medical Genetics and Genomics published guidelines in 2007 (updated in 2015), but these were and remain voluntary.^14,15^ Even when guidelines are voluntary, a court may still adopt them as a standard of care. In *Williams v. Athena*, however, the plaintiff does not rely solely on reference to regulation or guidelines; instead, she argues that Athena failed to follow its *own* classification scheme. Athena issued variant classifications along a 7-point scale in its reports, from benign to pathogenic. Two articles had previously been published identifying the variant as “pathogenic” (both relying on a single case and published before the June 2007 report was issued). When Athena issued an updated report in 2015 reclassifying Christian’s mutation as pathogenic, it cited no new evidence (and still failed to cite the 2006 and 2007 publications that reported the mutation). Does this lack of new evidence demonstrate that Athena negligently misclassified the variant as pathogenic in the original report? Other important but less generalizable allegations are that Athena held specific knowledge about this variant. One of the articles was coauthored by the laboratory director at Athena who signed-off on Christian’s test report.^18^ Patent submissions previously made by Bionomics (a company in Australia that licensed *SCN1A* testing rights to Athena) identified the mutation as pathogenic. This case is important for variant databases, which may perform many of the same interpretation activities as genetics laboratories. Prudent variant databases should ensure that such activities adhere to both voluntary best practice guidelines and database policies. Guidelines are emerging for databases, too. The Food and Drug Administration recently proposed voluntary standards and a recognition process for public variant databases as part of its flexible and adaptive approach to regulate next-generation sequencing tests.^10^ Test developers would be able to rely on recognized databases as evidence of the clinical validity of a next-generation sequencing test. Although this approach alleviates the challenge that developers face when amassing large amounts of variant data, it presents new concerns regarding how responsibility for data quality, transparency, and currency will be shared between test developers, variant databases, and their submitters.^19^

*Williams v. Athena* also illustrates how issues of interpretation can bleed into issues of communication. There is some controversy in the case regarding who received the test result and how it was communicated. Standards for reporting genetic results are another area of uncertainty, especially for variants of unknown significance.^16^ By analogy, variant databases should be careful about how they present variant data and should provide transparent information about data provenance and the processes used in interpretation. Finally, the relationship between interpretation and communication also raises issues about causation.^20^ In addition to fault, Williams must prove the misclassification *caused* premature death. This proof is hindered by the intervention of a physician (was it reasonable for Christian’s physician to base a treatment decision on the report?), which may break the chain of causation and scientific uncertainty (would a change of treatment have prevented the fatal seizure?).^21^ These uncertainties over causation are even more pronounced for misclassified data sharing through a variant database.

## Variant Databases: Addressing Liability Concerns

In light of *Williams v. Athena*, variant database administrators may have heightened concerns about liability. The scope of the legal duties of a variant database remains unclear. They are probably limited by the fact that submitters and users are both already subject to strict regulatory and professional duties. Interpretations submitted to a variant database are generated by laboratories under increasingly standardized and regulated conditions. Clinicians using variant databases are held to professional standards. They are expected to be up to date about the state of knowledge and limits of the data in databases, as they are with test reports.^20^ They continue to have a duty to interpret a variant classification in the context of an individual patient, e.g., by reviewing the literature, taking into account family history, or ordering follow-up tests for family members. In addition, courts have rarely found that authors or publishers have legal duties toward readers (including on the Internet). Courts seem more likely to find a duty when physical harm results. However, courts are less likely to find a legal duty when there is no contractual relationship, reliance on the information is not justified, and when information sharing supports policies of innovation and free expression.^22^

A database performing expert curation and classification does seem more likely to be liable for negligent interpretation than a database acting as a mere conduit. The Public Research Space of the BRCA Exchange, for example, provides public access to *BRCA* variant calls and classifications that are as comprehensive as possible and drawn from many databases. The variants reported will have been classified by someone, often an expert panel, such as a laboratory-associated expert team (such as the GeneDx or InVitae or Ambry team) or one of the national or regional *BRCA* testing laboratories. In these cases, the database should make the provenance of the variant data clear and clarify that the responsibility for the interpretation rests primarily with the submitting laboratory or database. In contrast, the BRCA Exchange Consensus Space “shows variants curated and classified by an international expert panel, the ENIGMA consortium, to assess their pathogenicity (associated disease risk).” This information is presented as more reliable.

**Figure 1:**
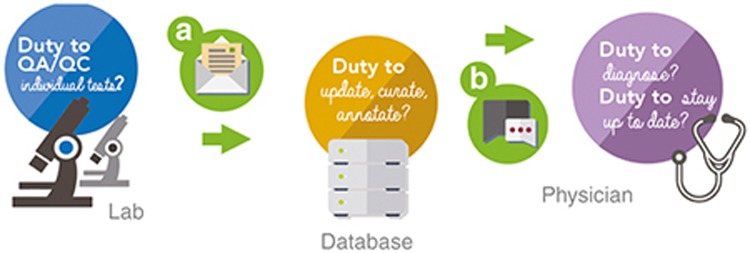
**Tri-partite responsibilities for genomic data sharing.** Data sharing in clinical genomics blurs the legal duties of laboratories, public variant databases, and physicians. To manage legal risk, public variant databases can establish a governance framework to clarify respective responsibilities, including (**a**) submission agreements and (**b**) terms of use/disclaimers.

Variant databases can mitigate legal risk by enhancing the accountability of submitters and users. This can be achieved, in part, with clear site descriptions, submission agreements, and terms of use agreements (see **Figure 1**).

### Site descriptions

The purpose of and intended audience for a database should be clearly established and prominently displayed.^8^ The BRCA Exchange describes its aim to “Create a curated list of BRCA variants, interpreted by expert consensus, to enable, without dictating, accurate clinical care.” Transparent descriptions of the data provenance and quality are also important representations from a liability perspective.

### Submission agreements

Variant databases share classifications published in the scientific literature as well as classifications submitted directly by clinical laboratories. Direct submission of variant data by clinical laboratories makes data available that would not otherwise reach the public domain. In such cases, variant databases should establish a submission policy or submission agreement to clarify respective responsibilities. The pros of submission agreements are that they can impose contractual accountability on submitters for the quality of data and for meeting legal requirements and ethical standards. The ClinVar database submission policy, for example, requires classifications to be made according to a comprehensive review of evidence consistent with, or more thorough than, current practice guidelines.^23^ A submission click-wrap agreement requires submitters to attest that they have the right to submit data for unrestricted access and that the data are accurate. Database managers should be aware of the limits of these agreements. Strict submission requirements may discourage the voluntary submission of data. They may also shift responsibility to the database to hold submitters accountable for meeting the requirements.

### Terms of use/disclaimers

Terms of use clarify that users are expected to behave “as learned intermediaries, exercising customary clinical discretion and consulting other sources of relevant information.”^24^ Common elements include the following:

- Liability disclaimers: exclude liability for harm resulting from reliance on inaccurate information. Legal disclaimers are found in a variety of medical contexts. When liability cannot be disclaimed, user agreements may instead aim to limit liability or remedies.
- Indemnities: users agree to indemnify the database for costs or liability stemming from the user’s actions.
- No warranty: the database makes no representations, warranties, or assurances that the data are accurate, current, or fit for purpose (e.g., diagnosis).
- No medical advice: the user agrees to not make diagnostic decisions based solely on the information in the repository without consulting a health-care professional with the relevant expertise.

The Human Variome project has developed a standard text for database disclaimers based on common elements in existing disclaimers. It notifies users of appropriate uses and includes language to limit the liability of submitters, curators, and managers.^25^ The effectiveness of user agreements to modify the obligations of clinicians, who are the primary audience for variant databases, may be limited. Clinicians are already held to professional standards and are expected to be up to date with the current state of knowledge (and its limitations). The validity of liability disclaimers may also be uncertain, particularly when data are made available online and internationally. In many jurisdictions, there are limits on liability disclaimers (e.g., it may be illegal to disclaim liability for recklessness or negligence resulting in bodily harm). In addition, a simple notification on a public website may not be sufficient to form a legal agreement; some form of click-wrap/click-through agreement may be required. Even when use agreements have a limited impact on legal obligations, they are still important to clarify the respective responsibilities of submitters, databases, and users.

## Conclusion

High-quality databases linking genotypes and phenotypes will only grow in importance as next-generation sequencing is adopted in the clinic.^26^ Voluntary data sharing through public variant databases is an important means of improving the consistency of variant interpretation by genetics laboratories.^27^ It reveals discrepancies in interpretation, prompts refinement of methods, and reduces harmful delays or errors in diagnosis.^28^ A clear understanding of the evolving ethical and legal duties of laboratories, databases, and physicians will be crucial to the success of genomic data sharing. Site descriptions, submission agreements, and terms of use agreements are important tools for databases to communicate these respective responsibilities.

## Disclosure

The authors declare no conflict of interest.
